# Initial psychometric properties of an Arabic version of the disordered eating attitudes in pregnancy scale (A-DEAPS) among Lebanese pregnant women

**DOI:** 10.1186/s40337-022-00710-x

**Published:** 2022-11-21

**Authors:** Sarah Gerges, Sahar Obeid, Souheil Hallit

**Affiliations:** 1 School of Medicine and Medical Sciences, Holy Spirit University of Kaslik, P.O. Box 446, Jounieh, Lebanon; 2 Social and Education Sciences Department, School of Arts and Sciences, Lebanese American University, Jbeil, Lebanon; 3 Research Department, Psychiatric Hospital of the Cross, Jal-Eddib, Lebanon; 4 Applied Science Research Center, Applied Science Private University, Amman, Jordan

**Keywords:** Eating disorder, Pregorexia, Disordered eating attitudes in pregnancy scale, Pregnancy, Lebanon, Psychometric properties, Cultural adaptation

## Abstract

**Background:**

Pregorexia refers to the excessive fear of pregnancy-induced weight gain and the drive to control it through various measures (e.g., extreme restriction of calorie intake, excessive exercising, or diuretics and/or laxatives consumption). The Disordered Eating Attitudes in Pregnancy Scale (DEAPS, Bannatyne et al., in: Disordered eating in pregnancy: the development and validation of a pregnancy-specific screening instrument. Bond University, 2018) is a brief pregnancy-specific instrument developed to screen for antenatal eating disorders. Our study’s objective was to examine the reliability and psychometric properties of the Arabic version of this pregnancy-specific scale among Lebanese pregnant women.

**Methods:**

We conceived and implemented a cross-sectional survey between June and July 2021 (N = 433). The sample was randomly divided in two as per the SPSS data selection option; the first subsample was used to conduct the DEAPS items’ exploratory factor analysis (EFA), whereas the second was used for the confirmatory factor analysis (CFA). Within this study, we described multiple indices of goodness-of-fit: the Relative Chi-square (χ^2^/df), Root Mean Square Error of Approximation (RMSEA), Tucker Lewis Index (TLI), and Comparative Fit Index (CFI).

**Results:**

An EFA was conducted on subsample 1 (N = 207), chosen randomly from the original sample. With the exception of item 8, all other 13 items converged over a two-factor solution [Factor 1 (3 items): Body Image Concerns during Pregnancy, and Factor 2 (10 items): Disordered Eating Attitudes during Pregnancy]. In subsample 2 (N = 226), the CFA results showed that the one-factor model (Factor 2: 10 items), which derived from the EFA conducted on subsample 1, fitted well accordingly to CFI, TLI, and χ^2^/df values, and fitted modestly according to RMSEA. The CFA estimates obtained for model 1 (original scale of 14 items) and model 2 (according to the two-factor solution obtained from the EFA in subsample 1) fitted less than the third model (Factor 2). The analysis thus suggested retaining only Factor 2 with 10 items in the Arabic version of the scale.

**Conclusion:**

Our study was able to provide preliminary evidence that the Arabic 10-item version of the DEAPS seems to be a good and reliable tool for the assessment of disordered eating attitudes among Lebanese pregnant women.

**Supplementary Information:**

The online version contains supplementary material available at 10.1186/s40337-022-00710-x.

## Plain English summary

Pregnancy remains a complex “biopsychosocial phenomenon” that can witness the emergence of concerns about weight, body image, femininity, and self-esteem. Pregorexia refers to the excessive fear of pregnancy-induced weight gain and the drive to control it through various measures (e.g., extreme restriction of calorie intake, excessive exercising, or diuretics and/or laxatives consumption). The Disordered Eating Attitudes in Pregnancy Scale (DEAPS, Bannatyne et al., in: Disordered eating in pregnancy: the development and validation of a pregnancy-specific screening instrument. Bond University, 2018) is a brief pregnancy-specific instrument developed to screen for antenatal eating disorders. Our study's objective was to examine the reliability and psychometric properties of the Arabic version of this pregnancy-specific scale among Lebanese pregnant women. Ultimately, our study was able to provide preliminary evidence that the Arabic 10-item version of the DEAPS seems to be a good and reliable tool for the assessment of disordered eating attitudes among Lebanese pregnant women.

**Supplementary Information:**

The online version contains supplementary material available at 10.1186/s40337-022-00710-x.

## Background

Lately, there has been an upsurge in the prevalence of eating disorders, owing primarily to changes in sociocultural factors [1]. Body image disorders are not benign. They dictate risky behaviors that engender major physical and mental health harm as well as damage to social life [1]. Through the history of mankind, the concept of beauty has evolved to the point that thinness nowadays represents success among women. It is estimated that 11–72% of them are dissatisfied with their body [2].

Pregnancy remains a complex “biopsychosocial phenomenon” that can witness the emergence of concerns about weight, body image, femininity, and self-esteem [3]. These preoccupations are usually triggered by the emotional-hormonal ambivalence of pregnancy, resulting in the development of new eating disorders or the exacerbation of previously existing ones as a means of coping with harmful feelings such as anxiety or phobic and obsessive–compulsive traits [3]. Consequently, pregnancy may serve as a basis for the occurrence of “pregorexia”—a notion of popular psychology designating a newly emerging behavior. In 2008, the term “pregorexia” first appeared in “The Early Show and Fox” press, referring to the excessive fear of pregnancy-induced weight gain and the drive to control it through various measures (e.g., extreme restriction of calorie intake, excessive exercising, or diuretics and/or laxatives consumption) [4, 5].

This neologism results from combining “pregnancy” and “anorexia” [5]. However, to date, “pregorexia” has neither been considered a medical diagnosis nor classified in the Diagnostic and Statistical Manual of Mental Disorders, Fifth Edition (DSM-5) eating disorders criteria. Additionally, it has not been assigned any international formal or medical definition yet [6]. We might simply define it as anorexia nervosa occurring for the first time during pregnancy [5], taking into account that women with “pregorexia” may experience eating restriction as well as bingeing followed by purging [4]. In addition, some alarming signs or “red flags” for “pregorexia” have been identified, such as skipping meals, eating alone, and talking about pregnancy as if it is not real (i.e., state of denial) [4].

Surprisingly, the prevalence of eating disorders (i.e., anorexia nervosa, bulimia nervosa, binge eating disorders, Other Specified Feeding or Eating Disorder, etc.) during pregnancy varies greatly between studies, ranging from 0.6 to 27.8% [7–18]. These disparities could be explained by the diversity of assessment tools, varying from self-report questionnaires to structured interviews [8]. Additionally, the self-report measures used in research were inconsistent with one another, as some tools were based on adapted versions of pre-existing scales for eating disorders, whereas others were designed on items derived from the DSM-4/DSM-5 criteria [8]. This incongruity has therefore posed fundamental problems in the assessment of disordered eating during pregnancy, emphasizing the need for a consensual and accurate pregnancy-specific screening tool, as previously suggested by a Delphi study [19], to facilitate comparisons in research and reduce the likelihood of false negative and false positive tests.

Furthermore, a recent systematic review has refuted the suitability of traditional existing measures for detecting eating disorders in pregnancy, pointing out that only four of sixteen scales used across countries were presented with established psychometric properties [18]. Namely, the Eating Disorder Examination (EDE), a semi-structured clinical interview, and three self-report measures, the Eating Disorder Examination Questionnaire (EDE-Q) [20, 21], the Eating Disorders Inventory-2 (EDI-2) [22], and the Disordered Eating Behavior Scale (DEBS) [23], have been used among pregnant samples [18]. Nonetheless, none of these instruments was able to show a commendable degree of clinical pertinence in terms of psychometric performance, such as internal consistency, criterion-related validity, or screening accuracy. As a result, no existing scale could be set as a “gold standard” measure or substitute the necessity for a specially designed instrument to identify dysfunctional eating symptoms during pregnancy [18].

Besides, when it comes to “pregorexia”, to the best of our knowledge, the only documented prevalence worldwide is 5% [24]. Nevertheless, most healthcare professionals are unaware of this condition [6]. The scarcity of studies exploring this phenomenon proves that raising awareness is essential, especially when considering the importance of a balanced diet during pregnancy and the risks of undernutrition for both the mother and fetus (e.g., placental abruption, miscarriage, low birth weight, type 2 diabetes mellitus, cardiovascular diseases, neural tube defects, cognitive disorders, as well as maternal anemia, impaired bone mineralization, post-partum depression, etc.) [25, 26]. In fact, barriers to the identification of eating disorders during pregnancy are principally stigma and poor professional training [27]. In addition, the lack of confidential discussions about weight gain, mental health, and body dissatisfaction between pregnant women and their physicians accounts for the limited detection and management of “pregorexia” [4].

Furthermore, the literature has highlighted the associations between disordered eating during pregnancy and maternal psychological distress (i.e., anxiety, stress, and depressive symptoms) [15, 28], accentuating the threatening impact of these conditions on maternal mental health. In light of these facts, providing healthcare professionals with an efficient screening tool for the early detection of disordered eating attitudes during pregnancy is of prime importance, in order to optimize diagnostic and treatment procedures and thus circumvent negative health repercussions. However, it is only in 2018 that Bannatyne et al. generated a brief pregnancy-specific instrument in furtherance of screening for antenatal eating disorders: the Disordered Eating Attitudes in Pregnancy Scale (DEAPS), which demonstrated a high level of internal consistency (Cronbach’s alpha value of 0.85) and good validity (KMO value of 0.88; *p* < 0.001) [16]. The DEAPS also had a unidimensional latent structure and a strong correlation with EDE-Q, supporting its convergent validity and further strengthening its construct validity [16]. This scale’s items were built on the results of their authors’ Delphi study, which made the distinction between anodyne pregnancy-related changes in eating habits and pathological eating symptomatology during pregnancy [29]. In order to be applied among Lebanese pregnant women, its cross-cultural adaptation requires a translation process into Arabic, Lebanon’s native language. Therefore, our study's objective was to examine the reliability and psychometric properties of the Arabic version of this pregnancy-specific scale among Lebanese pregnant women.

## Methods

### Participants

The sample consisted of 433 pregnant women aged between 19 and 43 years (Age range = 24; Mage = 28.55; SD = 4.63 years) with a mean gestational age of 23.68 ± 8.68 weeks. Other characteristics and description of the total sample, subsample 1, and subsample 2 can be found in Table 1 (Table 1).

**Table 1:** Sociodemographic characteristics of the participants

Variable	Total sample (N = 433)	Subsample 1 (N = 207)	Subsample 2 (N = 226)
*Education level*			
Secondary or less	62 (14.3%)	29 (14.0%)	33 (14.6%)
University	371 (85.7%)	178 (86.0%)	193 (85.4%)
*Marital status*			
Married	433 (100%)	207 (100%)	226 (100%)
*Religion*			
Christian	107 (24.7%)	51 (24.6%)	56 (24.8%)
Muslim	326 (75.3%)	156 (75.4%)	170 (75.2%)

	Mean ± SD	Mean ± SD	Mean ± SD
Age (in years)	28.55 ± 4.63	28.43 ± 4.53	28.66 ± 4.73
Household crowding index	0.82 ± 0.44	0.81 ± 0.50	0.83 ± 0.39
Physical activity index	12.08 ± 14.48	12.46 ± 13.74	11.72 ± 15.15

### Questionnaire and variables

Our questionnaire was administered in the Arabic language and consisted of closed-ended questions requiring about 15 min to be achieved. The first section collected socio-demographic characteristics, including age, marital status, religion, educational level (i.e., complementary, secondary, or university), and the household crowding index. The latter was determined by computing the ratio: number of people living in the respondent’s house/number of rooms in the house (except for the kitchen and bathrooms). Higher ratios are indicative of lower socioeconomic status [30]. The intensity, duration, and frequency of physical activity during pregnancy were multiplied to calculate the physical activity index [31].

The second part of the questionnaire included different scales:

*The Disordered Eating Attitudes in Pregnancy Scale (DEAPS)*: It is a brief measure, composed of 14 dichotomous items (e.g., “I have attempted to stop the changes occurring to my body during pregnancy” and “I have spent considerable time researching the most effective ways to minimize how much weight I gain while pregnant”) scored as a Yes/No type of answer. It was purposefully developed and validated to screen for pregnancy-specific symptoms of disordered eating [16]. Actually, researchers have endorsed that physicians should employ brief and psychometrically satisfactory instruments to detect medical problems in overwhelmed clinical settings [32], specifically during antenatal care [18]. The higher the score, the stronger the disordered eating attitudes during pregnancy [16].

*The Restraint Scale of the Dutch Eating Behavior Questionnaire (DEBQ-R)*: It is a concise instrument including 10 items scored on a 5-point Likert scale (ranging from 1 = “never” to 5 = “always”). Items assess the frequency of dietary restraint (e.g., “When you have put on weight, do you eat less than you usually do?”, “Do you try to eat less at meal times than you would like to eat?”, and “Do you deliberately eat less in order not to become heavier?”). Higher scores reflect higher trends of restrained eating [33], with good reliability and validity in multiple populations and settings [34, 35]. This scale has also been validated in the Arabic language in Lebanon [36, 37]. In this study, the Cronbach’s alpha = 0.93.

*The SCOFF questionnaire*: The SCOFF (**S**ick “Do you make yourself **S**ick because you feel uncomfortably full?”, Control “Do you worry you have lost Control over how much you eat?”, One “Have you recently lost more than One stone (6.53 kg) in a three-month period?”, Fat “Do you believe yourself to be Fat when others say you are too thin?”, Food “Would you say Food dominates your life?”) questionnaire is a short 5-item eating disorder screening tool [38], namely for anorexia and/or bulimia. These five questions have a Yes/No type of answers. The SCOFF overall appears to be the most frequently recommended method for identifying disordered eating during pregnancy [39], yet was not empirically validated in pregnant samples. The Arabic version of the SCOFF has been previously validated among Lebanese women [40]. In this study, the Cronbach’s alpha = 0.35. In fact, within the literature, the SCOFF's Cronbach's alpha values have been relatively low (i.e., below the threshold for acceptable internal consistency in diagnostic tests: lower than 0.7) [41–45], mainly due to the small number of its dichotomous items. These coefficients, however, can be considered acceptable for screening tests [46, 47].

### Study design

We conceived and implemented a cross-sectional survey between June and July 2021. An online self-administered, anonymous questionnaire, established on Google Forms, was disseminated to avoid the risk of face-to-face exposure during the COVID-19 pandemic. Pregnant women aged 18 years and above, from all the Lebanese districts/governorates (i.e., Beirut, Mount Lebanon, North Lebanon, South Lebanon, and Bekaa), were invited to take part in this study. They could access the link via social media platforms, such as WhatsApp and Facebook applications. Additional participation was guaranteed by the snowball technique, as all participants were requested to share the link among other pregnant women once completing the survey. In fact, online surveys have shown their efficacy in overcoming geographic distances and easing difficult access to specific populations [48] (e.g., pregnant women), hence providing a time-saving data collection at a national level.

### Minimal sample size calculation

A minimal sample consisting of 140 pregnant women was a requisite for the validation process, in reference to the recommendations of Comrey and Lee [49] who state that ten observations are mandatory for each item composing a scale (i.e., the fourteen items of the DEAPS).

### Translation procedure

In this study, the DEAPS was the sole scale needing to be validated in the Arabic language in Lebanon; hence, its cross-cultural adaptation was initiated by a two-step translation procedure: a forward translation (from English to Arabic), then a backward translation (from Arabic to English), performed by two distinct healthcare professionals, native Arabic speakers and fluent in English. The procedure was repeated until all the inconsistencies were solved. This translation process is in conformity to international guidelines required for a scale’s validation [50, 51]. The final Arabic version was approved by a committee constituted of two psychologists and two psychiatrists.

### Statistical analysis

The SPSS software v.25 was used for the data analysis. The option "required" was previously set for all questions on Google forms, in order to avoid missing data. Weighting to the general population was done according to the education level. The sample was randomly divided in two as per the SPSS data selection option; the first subsample was used to conduct the DEAPS items’ exploratory factor analysis (EFA), whereas the second was used for the confirmatory factor analysis (CFA). The FACTOR program was used to conduct the EFA; the EFA was initiated to confirm the legitimacy of the construct of the DEAPS in our sample. The principal component analysis was used as the method for component extraction; the Pearson correlation matrix was used during the analysis. The parallel analysis was used to determine the number of dimensions, with the normalized varimax used as a rotation to achieve factor simplicity. The Kaiser–Meyer–Olkin (KMO) value and the Bartlett's sphericity test were checked for sampling adequacy. The factors with Eigen values > 1 were kept. A Weighted Root Mean Square Residual (WRMR) < 1 represented a good fit [52]. Reliability was checked using the Cronbach's alpha for the total scale and its subscales. The SPSS AMOS software v.24 was used to conduct the CFA on subsample 2. The described indices of goodness-of-fit were: the Relative Chi-square (χ2/df) (cut-off values: < 2–5), the Root Mean Square Error of Approximation (RMSEA) (close and acceptable fit are considered for values < 0.05 and < 0.11 respectively), the Tucker Lewis Index (TLI), and the Comparative Fit Index (CFI) (acceptable values are ≥ 0.90) [53, 54].

The DEAPS score had a normal distribution, as the skewness and kurtosis values ranged between − 1 and + 1 [55]. These conditions reinforce the assumptions of normality in samples larger than 300 [56]. The Student t test and ANOVA F tests were used to compare two and three or more means, respectively. Pearson correlation test was used to correlate two continuous variables. A *p*-value < 0.05 was deemed statistically significant.

## Results

### Exploratory factor analysis

An EFA was conducted on subsample 1 (N = 207), chosen randomly from the original sample. The KMO value (= 0.841) and the p-value of the test of sphericity (X^2^(df) = 663.9(91); *p* < 0.001) confirmed the sample adequacy. With the exception of item 8, all other 13 items converged over a two-factor solution [Factor 1 (3 items): Body Image Concerns during Pregnancy, and Factor 2 (10 items): Disordered Eating Attitudes during Pregnancy], explaining a total variance of 40.5% (Table 2). The Weighted Root Mean Square Residual (WRMR) was 0.094 (95% CI 0.086–0.099).

**Table 2:** Exploratory factor analysis of the Disordered Eating Attitudes in Pregnancy Scale (DEAPS) using the varimax rotation on subsample 1

DEAPS Item	Factor 1: Body Image Concerns during Pregnancy	Factor 2: Disordered Eating Attitudes during Pregnancy
1. I have felt distressed about the changes to my body and/or eating habits during pregnancy		0.504
2. I have attempted to stop the changes occurring to my body during pregnancy		0.595
3. I have felt anxious about eating in general, or about eating certain foods		0.670
4. I have felt distressed after eating because of its effect on my weight and shape		0.623
5. I have noticed that what I allow myself to eat and how much I can eat is connected to rules and conditions		0.665
6. I felt like there were times when I lost control over my eating and/or body	0.618	
7. I worried that I have, or will, become ‘fat’ during pregnancy		0.572
8. I have felt distressed and uncomfortably full (i.e., like I am going to burst) after eating a large amount of food	–	–
9. I have spent considerable time researching the most effective ways to minimize how much weight I gain while pregnant		0.641
10. I have spent considerable time researching how I can rapidly lose weight after I have given birth		0.482
11. I have felt disgusted or ashamed with my pregnancy body	0.719	
12. My evaluation of my body shape, weight, or size during pregnancy has significantly influenced how worthy I believe I am as a mother or person	0.665	
13. I have wanted my pregnancy body to be small, like I am “just bump” (i.e., only my stomach appears to have grown, with no weight or shape changes to other areas of my body)		0.589
14. I have found myself frequently (at least once a week) comparing my weight, shape, size, or eating habits to other women		0.374
Percentage of variance explained	16.0	24.5
Cronbach’s alpha	0.71	0.80

### Factorial validity (confirmatory factor analysis of different models)

The fit indices of the three tested CFA models conducted on subsample 2 are presented in Table 3 (Table 3). As can be seen, the one-factor model (Factor 2: 10 items), which derived from the EFA conducted on subsample 1 in this study, fitted well accordingly to CFI, TLI, and χ2/df values, and fitted modestly according to RMSEA. The CFA estimates obtained for model 1 (original scale of 14 items) and model 2 (according to the two-factor solution obtained from the EFA in subsample 1) fitted less than the third model (Factor 2). The analysis thus suggested retaining only Factor 2 with 10 items in the Arabic version of the scale. Consequently, 4 items were removed from the Arabic version:

- Item 8, which did not load on any factor (“I have felt distressed and uncomfortably full (i.e., like I am going to burst) after eating a large amount of food”). This item could be perceived as binge eating, which does not concur with the bulimic and anorexic tendencies described in “pregorexia” pattern.
- The 3 items that loaded on Factor 1: item 6 (“I felt like there were times when I lost control over my eating/body”), item 11 (“I have felt disgusted or ashamed with my pregnancy body”), and item 12 (“My evaluation of my body shape, weight, or size during pregnancy has significantly influenced how worthy I believe I am as a mother or person”). These items are more related to body-image related concerns and self-esteem issues (e.g., loss of control over body, shame, disgust, and feeling unworthy); they do not effectively portray the drive for thinness and the bulimic/anorexic tendencies during pregnancy that characterize “pregorexia” pattern.

**Table 3:** Fit indices of the three tested confirmatory factor analysis models of the Disordered Eating Attitudes in Pregnancy Scale (DEAPS)

	χ2(df)	*P*	TLI	CFI	RMSEA	90%CI	Factor correlation range
Model 1	281.06 (77)	< .001	0.809	0.839	0.08	0.07–0.09	0.28–0.75
Model 2	132.35 (64)	< .001	0.879	0.900	0.07	0.05–0.08	0.40–0.76
Model 3	75.36 (35)	< .001	0.904	0.926	0.07	0.05–0.09	0.42–0.75

Model 1 = one factor (all items) according to the original validation of the scale; Model 2 = two factors (without item 8) according to the exploratory factor analysis results obtained in subsample 1; Model 3 = one factor (10 items) after removing 4 questions not related to disordered eating attitudes during pregnancy (that are observed in “pregorexia”)

As a result, the 10 items that loaded on Factor 2 were kept in the Arabic version of the scale (items 1–5, 7, 9, 10, 13, and 14; refer to Table 2):

- Item 1. Distressed about changes to body/eating in pregnancy.
- Item 2. Desire/Attempts to stop bodily changes during pregnancy.
- Item 3. Anxious about eating.
- Item 4. Distressed after eating.
- Item 5. Rules and conditions connected to eating.
- Item 7. Fear of fatness in pregnancy.
- Item 9. Researching pregnancy weight loss.
- Item 10. Researching rapid postpartum weight loss.
- Item 13. Desire for small pregnancy body.
- Item 14. Comparison of weight/shape/size/eating in pregnancy with other pregnant women.

These items measure the desire to remain thin during pregnancy, the fear of pregnancy-related weight gain, and the different attempts to control/restrict this weight gain. Given the good validity (CFA results) and high internal consistency (Cronbach’s alpha = 0.8) of this one-factor model, only one component (Factor 2) was retained in the final Arabic version of the scale, which was suggested to have a unidimensional structure. The final Arabic version (A-DEAPS) can be found as a Additional file 1: Appendix 1.

### Convergent validity

Higher A-DEAPS scores correlated well with higher restrained eating (r = 0.593; *p* < 0.001) and higher SCOFF (r = 0.503; *p* < 0.001) scores in the total sample.

### Correlation of each item of the A-DEAPS (i.e., the newly generated 10-item scale) with the A-DEAPS total score

Each item of the newly generated A-DEAPS (i.e., items 1–5, 7, 9, 10, 13, and 14) correlated well with the 10-item A-DEAPS total score, with correlation coefficients varying between 0.460 and 0.751.

## Discussion

### Factorial validity of a new one-factor model of the DEAPS

In this paper, we inspected the psychometric properties of the Arabic version of the DEAPS in a sample of Lebanese pregnant women; this would help surmount language barriers and properly screen for “pregorexia”. The authors who originally developed the DEAPS suggested a one-factor model for the scale, and refined it to 14 items to support their one-component model [16]. Through this study, the EFA results yielded a two-factor solution for the Arabic version of the scale, with the exception of item 8 (i.e., perceived loss of control over eating). In the CFA, we thus tested three measurement models for the Arabic version; the CFA results of the original model (14-item version) and the two-factor model [Factor 1: Body Image Concerns during Pregnancy (3 items), and Factor 2: Disordered Eating Attitudes during Pregnancy (10 items)] showed poor fitting results.

However, the CFA results of the third model, taking Factor 2 (10 items; refer to Table 2) alone, came out to be more satisfactory. In fact, the 10 items that loaded on Factor 2 assess disordered eating attitudes in pregnancy (i.e., fear of gaining weight during pregnancy, drive for thinness, and weight restriction observed in “pregorexia”), while Factor 1 contains items that describe body image-related concerns and self-esteem issues during pregnancy (i.e., feeling “out of control” of one’s body, feelings of shame and disgust about body, and feelings of unworthiness). Consequently, our study suggested that the one-factorial model (Factor 2), which includes 10 items only, shall preferably be considered regarding the A-DEAPS. As a result, we removed item 8 that corresponds to binge eating and Factor 1 (items 6, 11, and 12) from the Arabic version of the scale.

### Reliability and convergent validity of the new one-factor model

The 10-item, one-factor model seems to be internally consistent, and the reliability analysis of these 10 items in the total sample turned out to be very good as well (αCronbach = 0.87). This value was similar to the one obtained in the original version of the DEAPS (αCronbach = 0.85) [16]. In addition, the item-total correlation appeared to be good, suggesting that the chosen items represent the A-DEAPS well. Furthermore, the A-DEAPS correlated well with the restrained eating and SCOFF scores, demonstrating the convergent validity of the scale. All these findings suggest preliminarily good reliability and construct validity for the newly generated 10-item A-DEAPS.

### Limitations and strengths

Our study presents some limitations, such as its cross-sectional design. In addition, the snowball technique followed during the data collection process predisposes us to a selection bias. The symptoms of disordered eating were self-reported by the participants and not clinically diagnosed by a healthcare professional, making our results susceptible to a possible information bias. Residual confounding bias is also possible since other factors associated with disordered eating (e.g., mental health issues, smoking, etc.) were not taken into account in this paper. In addition, very few questionnaires were administered, limiting the capacity of the study to assess construct validity: For the convergent validity, we used two scales only (i.e., the DEBQ-R and the SCOFF questionnaire). Further studies focusing on other psychometric properties of the DEAPS (e.g., test–retest, convergent and divergent validity with other scales, etc.) should be conducted. Despite these limitations, our study provided the first suitable Arabic instrument for screening for disordered eating attitudes during pregnancy, which could aid in the implementation of systematic antenatal screening programs for eating disorders in Lebanon, easing pregnant women’s support and referral to a specialist as needed.

## Conclusion

In sum, our study was able to provide preliminary evidence that the Arabic 10-item version of the DEAPS seems to be a good and reliable tool for the assessment of disordered eating attitudes among Lebanese pregnant women. Further research should aim at validating this scale in the clinical setting and among other Arabic-speaking populations.

## Supplementary Information

**Additional file 1: Appendix 1.** Items of the Arabic version of the Disordered Eating Attitudes in Pregnancy Scale (A-DEAPS)

## Data Availability

The datasets generated and/or analyzed during the current study are not publicly available due to restrictions from the ethics committee but are available from the corresponding author on reasonable request.

## Abbreviations

DSM: Diagnostic and statistical manual of mental disorders
DEAPS: Disordered eating attitudes in pregnancy scale
DEBQ-R: Restraint scale of the Dutch eating behavior questionnaire
EFA: Exploratory factor analysis
CFA: Confirmatory factor analysis
RMSEA: Root mean square error of approximation
TLI: Tucker lewis index
CFI: Comparative fit index
