# Supplementary material for: Initial psychometric properties of an Arabic version of the disordered eating attitudes in pregnancy scale (A-DEAPS) among Lebanese pregnant women
Source: J Eat Disord. 2022 Nov 21;10:175. doi: 10.1186/s40337-022-00710-x (PMC9677897; doi:10.1186/s40337-022-00710-x)
Supplement: Supplementary file 1 — Additional file 1: Appendix 1. Items of the Arabic version of the Disordered Eating Attitudes in Pregnancy Scale (A-DEAPS) [file 40337_2022_710_MOESM1_ESM.docx]

Appendix 1. Items of the Arabic version of the Disordered Eating Attitudes in Pregnancy Scale (A-DEAPS)

1. لقد شعرت بالضيق بسبب التغييرات التي طرأت على جسدي و / أو على عادات أكلي أثناء الحمل
2. لقد حاولت إيقاف التغييرات التي تحدث في جسدي أثناء الحمل
3. لقد شعرت بالقلق بشأن تناول الطعام بشكل عام، أو بشأن تناول أطعمة معيّنة
4. لقد شعرت بالضيق بعد الأكل لما له من تأثير على وزني وشكلي
5. لقد لاحظت أنّ ما أسمح لنفسي بتناوله ومقدار ما يمكنني تناوله مرتبطَين بقواعد وشروط
6. قلقت بشأن أنّني أصبحت أو سأصبح "سمينةً" أثناء الحمل
7. لقد قضيت وقتًا طويلاً في البحث عن أكثر الطرق فعاليّة لتقليل مقدار الوزن الذي أكسبه أثناء الحمل
8. لقد قضيت وقتًا طويلاً في البحث عن كيفية فقدان الوزن بسرعة بعد الولادة
9. قد أردت أن يكون جسمي نحيفًا أثناء الحمل (أي أن يبدو أنّ بطني فقط قد نما، مع عدم تغيّر وزني وشكل مناطق أخرى من جسمي)
10. لقد وجدت نفسي بشكل متكرر (على الأقلّ مرّة في الأسبوع) أقارن وزني أو شكلي أو حجمي أو عاداتي بالأكل بنساء أخريات
